# Efficacy of Shexiang Tongxin Dropping Pills in a Swine Model of Coronary Slow Flow

**DOI:** 10.3389/fphys.2022.913399

**Published:** 2022-06-14

**Authors:** Yupeng Bai, Mingjing Zhang, Sheng Peng, Yuting Wang, Ye Gu, Qianqian Fang, Liqun Hu

**Affiliations:** ^1^ Department of Cardiology, Wuhan Fourth Hospital, Puai Hospital, Tongji Medical College, Huazhong University of Science and Technology, Wuhan, China; ^2^ Inner Mongolia Conba Pharmaceutical Co., Ltd., Hangzhou, China

**Keywords:** Shexiang Tongxin dropping pills, slow flow, swine, percutaneous coronary intervention, coronary microcirculation

## Abstract

**Objective:** Preliminary clinical studies have confirmed that Shexiang Tongxin dropping pills (STDPs) could improve angina pectoris and attenuate vascular endothelial dysfunction in patients with slow coronary flow, but the underlying mechanism is not fully unclear. We aimed to investigate the impact of STDP in a swine model of coronary slow flow (SF) and related mechanisms.

**Methods:** SF was induced by coronary injection of 40μm microspheres, and pigs were randomly divided into the SF group and SF plus STDP group. Pigs in the STDP group received sublingual STDP for 10 min, followed by 1 g STDP oral administration daily for 6 days. Coronary angiography was performed, the TIMI frame count (TFC) was determined, and hemodynamic measurements were performed before, at 30 min, and 7 days post-SF. Serum levels of total NO, NOS, ET-1, C-TNI, and BNP were measured. Myocardial expressions of TNF and IL-6, eNOS, VEGF, CD31, and α-SMA were analyzed by immunohistochemistry and Western blotting.

**Results:** Compared to the SF group, LVEF and TFC were significantly improved at 7 days post-SF in the STDP group. The serum ET-1 level was significantly reduced at 7 days, and NO and NOS levels were significantly higher in the STDP group. Seven days post-SF, myocardial TNF and IL-6 expressions were significantly downregulated, while the expressions of eNOS and VEGF, CD31, and ɑ-SMA were significantly upregulated in the STDP group.

**Conclusion:** Our results showed that STDP improved cardiac function and coronary flow, possibly through reducing inflammatory responses and upregulating myocardial eNOS and VEGF, CD31, and the ɑ-SMA expression**.**

## Introduction

Percutaneous coronary intervention (PCI) is one of the standard therapeutic options for patients with acute myocardial infarction (AMI) (Zimarino et al., 2008). Despite successful revascularization, the myocardial blood flow might still be comprised at the microvascular level in some patients; this “no-reflow/slow flow phenomenon” is largely induced by microemboli of atherosclerotic debris, spasm, microvascular damage, and thrombi generated during the PCI procedure (Niccoli et al., 2009), especially post-directional and rotational atherectomy. Clinical studies revealed that patients with the no-reflow phenomenon following reperfusion therapy for AMI faced a higher risk of worse outcomes than patients without the no-flow phenomenon (Herrmann, 2005; Heusch et al., 2009, 2018). Therefore, it is of great importance to achieve revascularization at the microcirculation level post-PCI (Lanza and Crea, 2010). At present, some progress has been made in the treatment of coronary microcirculation dysfunction; several drugs, such as sodium nitroprusside, adenosine, and verapamil, have shown beneficial effects on improving coronary non-reflow/slow flow (Barcin et al., 2004). Chinese traditional medicine was characterized by multi-component, multi-target, and multi-pathway. Shexiang Tongxin dropping pills (STDPs) were made up of artificial musk, total saponins from ginseng stems and leaves, and salvia miltiorrhize. Preliminary small-scale clinical studies confirmed that STDP could improve angina pectoris and attenuate vascular endothelial dysfunction in patients with slow coronary flow (Wang et al., 2019; Lu et al., 2020; Tian et al., 2020), but the underlying mechanism is not fully unclear. In this study, we observed the angiographic effects of STDP in a large animal (Bama pig) with a slow-flow model established in our laboratory (Hu et al., 2014; Bai et al., 2015) and observed its impact on serum and myocardial biomarkers related to inflammatory and angiogenetic pathways. We aimed to observe the effects of STDP by observing the coronary angiographic, hemodynamic, serum biomarkers, HE staining, and immunohistochemical staining changes in a swine model of coronary slow flow (SF) and explore the potential mechanisms related to these effects post-STDP treatment.

## Methods

### Experimental Animals and Shexiang Tongxin Dropping Pill Administration Protocol

A total of 18 male Bama miniature pigs (4 months old, 30 ± 2 kg) were purchased from Tongji Medical College, Huazhong University of Science and Technology, and kept in ordinary animal houses (Temperature: 15°C–25°C). Aspirin (2–3 mg/kg/d) was mixed in the food 3 days prior to experimental studies. The study protocol was approved by the Puai Hospital, Tongji Medical College Council on the Animal Care Committee of the Huazhong University of Science and Technology (Wuhan, China). Animals were maintained in accordance with the Guide for the Care and Use of Laboratory Animals published by the US National Institute of Health (NIH Publication No. 85-23, revised 1996). Twelve Bama miniature pigs underwent slow coronary flow operation and were randomly divided into the slow coronary flow group (underwent coronary injection of 40μm microspheres, SF group, *n* = 6) and the Shexiang Tongxin dropping pill group (underwent coronary injection of 40μm microspheres and performed STDP, STDP group, *n* = 6) by random number tables, and six animals were assigned to the Sham-operated group (SHAM group).

### Medication

STDP is a prescription medicine approved by the Chinese Food and Drug Administration (No. Z20080018), which consists of *Moschus*, Radix Rhizoma Ginseng, *Calculus bovis*, Fel Ursi, Venenum Bufonis, Borneolum syntheticum, and Salvia miltiorrhiza (Inner Mongolia Kangenbei Pharmaceutical Co., LTD.). Bama miniature pigs in the STDP group were treated with sublingual STDP powder immediately after the induction of slow flow. Briefly, STDP (1.4 g powder) was dissolved in 1.5 ml saline and mixed well, and the suspension was soaked in gauze strips; the gauze strips soaked with STDP were then placed under the tongue of pigs for 10 min. The pigs were resuscitated and kept in cages after the operation. The pigs in the STDP group were treated with 1 g STDP for 6 days. Briefly, 1 g STDP powder was mixed into 500 g pannage; after the pannage mixed with STDP was consumed each day, the remaining pannage was then added.

The applied concentrations of STDP powder in our study were referred to as the dose used in previous studies (Xiong et al., 2015; Lin et al., 2017). In the pilot study, we tested the effects of STDP at a dosage of 10 mg/kg/d, STDP 20 mg/kg/d, STDP 30 mg/kg/d, and STDP 40 mg/kg/d for 6 days (*n* = 1 each) on improving coronary flow in terms of the TIMI frame count (TFC) in comparison with the model group. As shown in Supplementary Table S1, STDP at a dosage of 30 mg/kg/d achieved expected effects on the 7th day, and the effects of STDP at a dosage of 40 mg/kg/d were similar to those of 30 mg/kg/d, so STDP 30 mg/kg/d was used for the main study. For sublingual STDP use for 10 min, the maximum amount of STDP powder that could be dissolved in 1.5 ml saline was 1.4 g, so this suspension was soaked in gauze strips; the gauze strips soaked with STDP were then placed under the tongue of pigs for 10 min.

### Slow Flow Model

**FIGURE 1:**
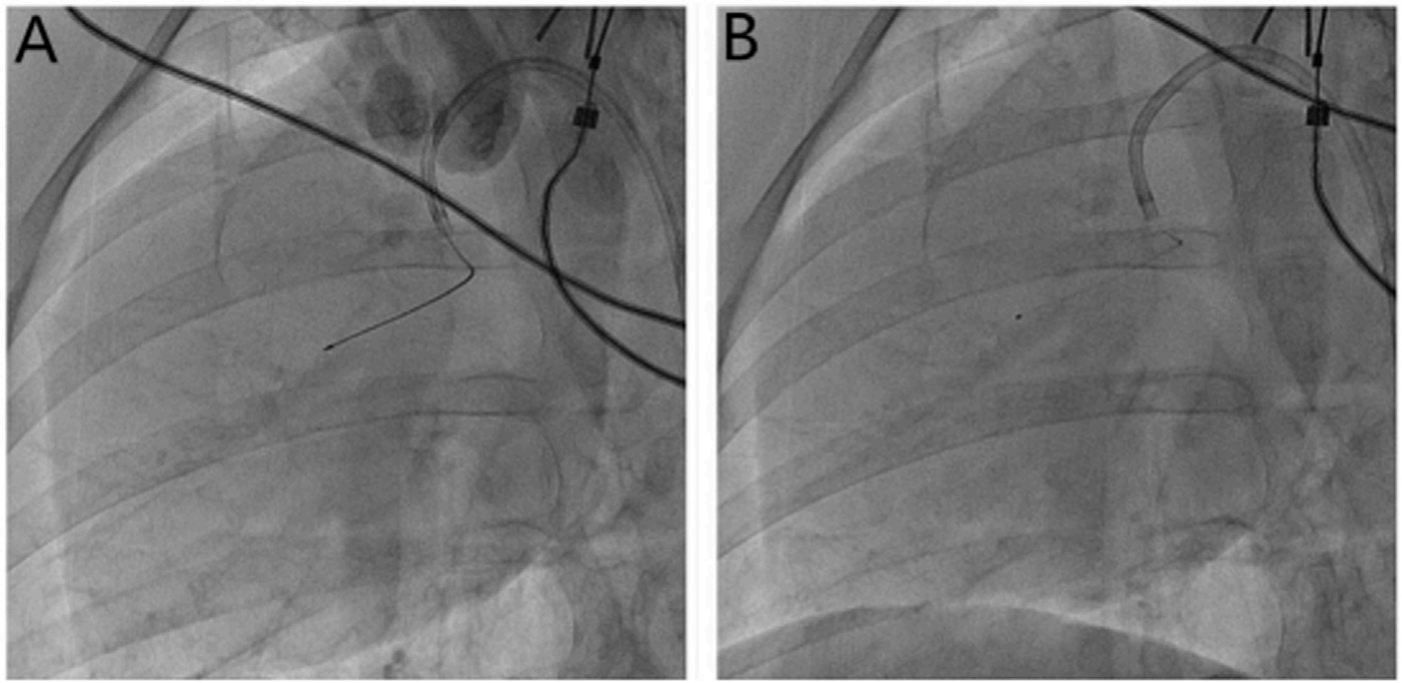
Microsphere injection method. **(A)** Measurement of the length of the anterior descending branch of the microcatheter (30 mm) with Maker; **(B)** the microcatheter is fixed, the guide wire is withdrawn, and the microspheres are injected through the microcatheter.

The method of the establishment of the SF model was similar to that previously reported (Hu et al., 2014; Bai et al., 2015). Briefly, pigs were fasted for 12 h before the operation and had free access to drinking water until 4 h prior to operation. Pigs were anesthetized by using an intramuscular injection of xylazine compound (xylazine hydrochloride, tiletamine hydrochloride, and midazolam as the active ingredients) combined with atropine (1 mg) and then fixed in a supine position on the workstation. An intravenous vein was quickly established in the ear vein of the Bama pig, and the intravenous vein was maintained with 5% glucose saline. During the operation, 0.1–0.2 ml of the xylazine compound was injected intramuscularly on demand to maintain the anesthesia state. ECG and vital signs were continuously monitored. Oxygen saturation (SO_2_) was measured with a pulse oximeter attached to the ear of pigs. Arrhythmias and anesthesia accidents were monitored. Blood samples were taken at baseline, 30 min, 24 h, and 7 days after the operation. Anticoagulation was induced with 200 IU/kg heparin sodium. The right femoral artery was dissected, and 6F vascular sheath was placed for arterial access. After initial coronary angiography (CAG) using a 6F JR3.5 guiding catheter (Medtronic, Inc.), ventriculography examinations, and LV pressure measurements obtained with a 5F Pigtail catheter (Cordis Inc.), a 2.6F infusion microtubule catheter (Terumo Corporation) was advanced to the middle part of the left anterior artery (LAD, around 30 mm from the LAD orifice) with the help of the guidewire (Whisper, Abbott Vascular) for microspheres or saline injection (Figure 1). Pigs in the SHAM group received repeated equal volume coronary saline injections. SF was induced and evaluated, as previously reported (Hu et al., 2014; Bai et al., 2015). In this experiment, we reduced the total amount of microsphere injections to further increase the survival rate. The injection dose used was 1.0 ml microsphere suspension (≈120,000 microspheres) to achieve an increase of the TFC ≥ 2 times the baseline level with the average TFC > 40 frames. Myocardial tissue perfusion was analyzed with the TMPG scale (Zietkiewicz et al., 1999) (TIMI myocardial perfusion grade). Coronary angiography (CAG) imaging (including TIMI grade, TFC, and TMPG grade) was measured at baseline, 1 min, 10 min, and 30 min after microsphere injection (Gibson et al., 1996; Wei et al., 2001; Poldervaart et al., 2017) The blood flow in the LAD was assessed with the TIMI scale and TFC, while myocardial tissue perfusion was analyzed with the TMPG scale (TIMI myocardial perfusion grade). Left ventricular (LV) end-diastolic volume and ejection fraction (LVEF), as well as LV pressure, were assessed at baseline and 30 min after successful modeling of SF. After retrieving the guidewire and catheter, the right femoral artery was sutured with 7.0 stylolite. The pigs were revived and kept for 7 days. Coronary angiography and left ventricular angiography were repeated on the 7th-day post-operation.

In our previous study, we observed the evolution of coronary flow in this swine model (Bai et al., 2015) here and found that coronary flow remained relatively stable at the 7th-day post microembolism, so we observed the impact of STDP medication up to 7 days on coronary flow in this model.

### Pathological Examination

On the 7th day after the operation, all 18 pigs were sacrificed by intravenous 10% KCl (potassium chloride) injection (30 ml) to stop the heart at the diastolic phase under deep anesthesia (intramuscular injection of 75 mg/kg ketamine). The heart was excised; right and left atria were removed, and the left ventricle (after separating the right ventricle) was washed with saline and weighed. After that, 500 mg of left ventricular (LV) apex tissue was obtained and divided into 5 parts and frozen in liquid nitrogen for Western blot analysis. The LV free wall near the LV apex was cut into 0.5 cm × 0.5 cm block and fixed in 10% formalin for 3 days, and then, it was dehydrated, paraffin-embedded, and cut into 4 μm sections for hematoxylin and eosin (HE) staining and immunohistochemistry examinations. Apoptosis staining using the EZClick™ TUNEL–in situ DNA Fragmentation/Apoptosis Assay Kit was performed on myocardial tissue derived from the LV free wall. For each animal, five random pictures were obtained at 200x magnification, and the results were analyzed by using a microscope connected with a computer and microscopic image analysis system (Image Pro-4, Media Cybernetics, Inc., Atlanta, GA). In HE-stained sections, the percentage of micro thrombosis was determined to analyze the inflammatory response; the inflammatory cells (leukomonocytes) were counted in 10 randomly selected visual fields in each section and five sections per pig.

### Determination of Serum Markers

Blood (10 ml) was collected *via* femoral vein at baseline, 30 min, 24 h, and 7 days after microsphere injection from animals in each group and centrifuged, and serum was stored at −80°C. Total nitric oxide (NO), NO synthase (Nanjing Jiancheng Institute of Biology), and ET-1 (R & D SYSTEMS, Endothelin-1 Quantikine ELISA Kit) were determined at baseline, 30 min, and 7 days after the operation, and cardiac troponin I (c-TNI) and brain natriuretic peptide (BNP) were determined at baseline, 24 h and 7 days by using respective ELISA kits (Wuhan Elabscience Biotechnology Co., Ltd.), according to the manufacturer’s instruction.

### Western Blot Analysis

Protein expressions of eNOS, VEGF, CD31, α-SMA, tumor necrosis factor-alpha (TNF-α), and interleukin 6 (IL-6) were determined by Western blot analysis on the left ventricular apex obtained from sacrificed animals 7 days after the operation. Tissues were homogenized in PBS and centrifuged at 10,000 × g for 10 min at 4°C, and then, the supernatant was lysed in electrophoresis buffer, boiled for 10 min, and subsequently subjected to electrophoresis on an SDS polyacrylamide gel. The separated blots were transferred to nitrocellulose membranes and blocked for 1 h in a TBS buffer containing 5% nonfat milk. The membranes were incubated overnight with primary antibodies: TNF-alpha (Abcam, ab1793,1:500), IL-6 (Bioworld, BS6419,1:500), eNOS (Abcam, ab5589,1:500), VEGF (Immunoway, YT4870,1:500), CD31 (GeneTex, GTX42089,1:500), α-SMA (GeneTex, GTX100095,1:1000), and secondary antibody (Goat-anti-rabbit, Goat-anti-mouse; KPL, Milford, MA, United States), and coloration, immunoreactive bands were obtained. Blots were detected by chemiluminescence, and the relative protein expression was quantified by scanning densitometry.

### Statistical Analyses

The data are analyzed using SPSS 12.0 (SPSS, Inc., Chicago, IL, United States). Data are presented as the mean ± SD. Differences in mean values between the various groups and between various times were compared by two-way ANOVA, followed by the Student–Newman–Keuls *post hoc* test. *p* < 0.05 was considered statistically significant.

## Results

### Serial Changes of Serum Biomarkers

**FIGURE 2:**
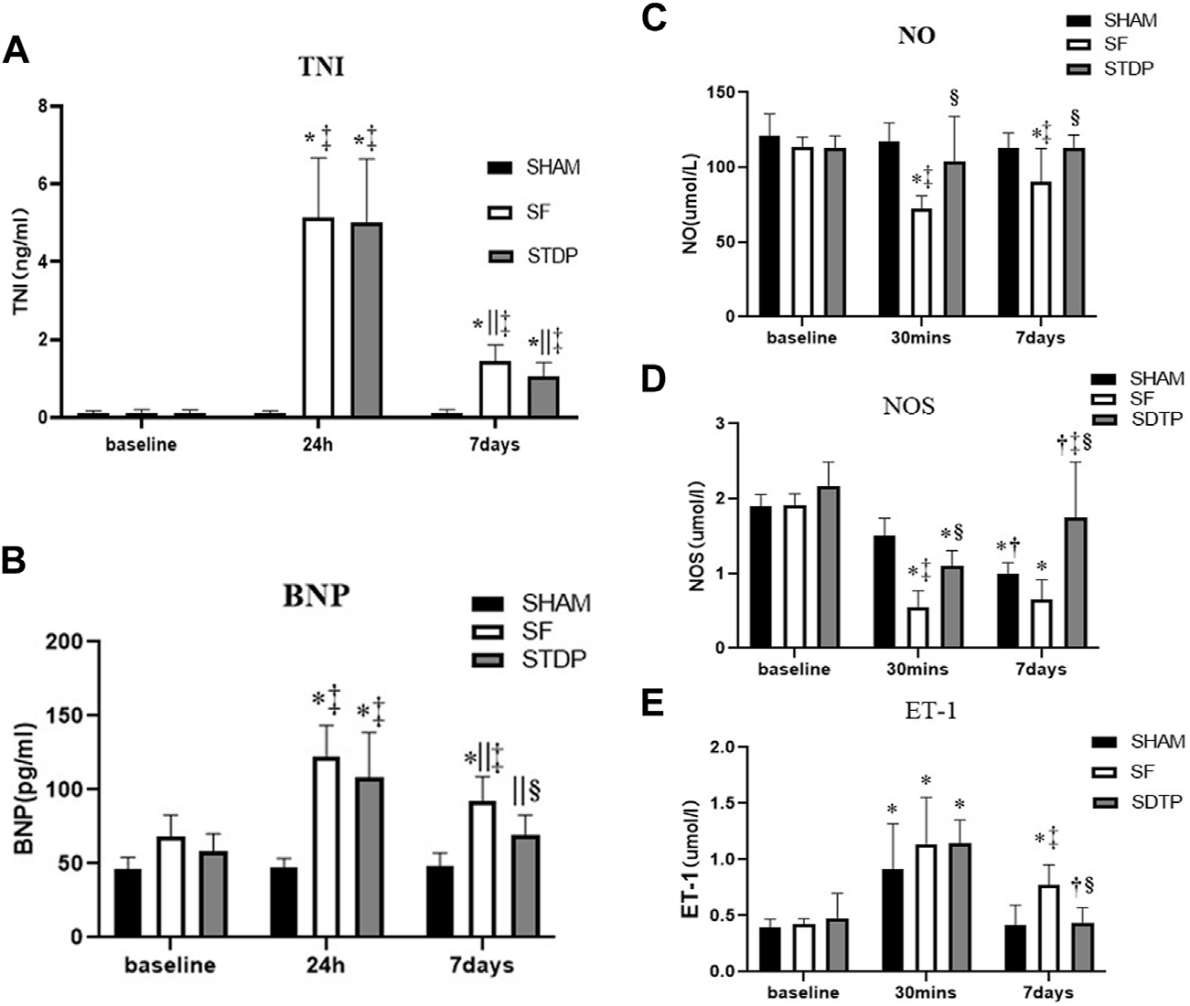
Serum c-TNI, BNP, NO, NOS, and ET-1 levels. **(A)** Serum c-TNI level; **(B)** serum BNP level; **(C)** serum total NO level; **(D)** serum NOS level; **(E)** serum ET-1 level. Data are presented as the mean ± SD, *p* < 0.05 *vs. baseline, †vs. 30 min, ||vs. 24 h, ‡vs. Sham group, and §vs. SF group (*n* = 6 for each group).

The serum TNI level was significantly higher at 24 h and 7 days after embolization than that before surgery and not affected by STDP (Figure 2A). The serum BNP level was significantly higher at 24 h and 7 days after embolization than before surgery, which was significantly lower in the STDP group than in the SF group at 7 days post-embolization (Figure 2B). Nitric oxide (NO) is considered an endogenous vasodilator and an important marker of endothelial function. NOS is the key enzyme responsible for the production of NO in vascular endothelial cells (Beltrame et al., 2002; With Notø et al., 2006; Flammer et al., 2012; Folino et al., 2013). Serum levels of NO and NOS decreased significantly at 30 min and 7 days in the SF group compared to the SHAM group, NO was significantly higher in the STDP group than in the SF group at 7 days post embolization, and NOS was significantly higher in the STDP group than in the SF group at 30 min and 7 days after embolization (Figures 2C,D). Endothelin-1 (ET-1) is a peptide produced by the endothelium of blood vessels that causes vasoconstriction (Stewart et al., 1991; Tønnessen et al., 1995). Compared with the SHAM group, the serum ET-1 level in the SF group was significantly increased at 30 min and recovered after 7 days after embolization. Compared with the SF group, the ET-1 level was significantly lower at 7 days post embolization (Figure 2E).

### TIMI Frame Count

**FIGURE 3:**
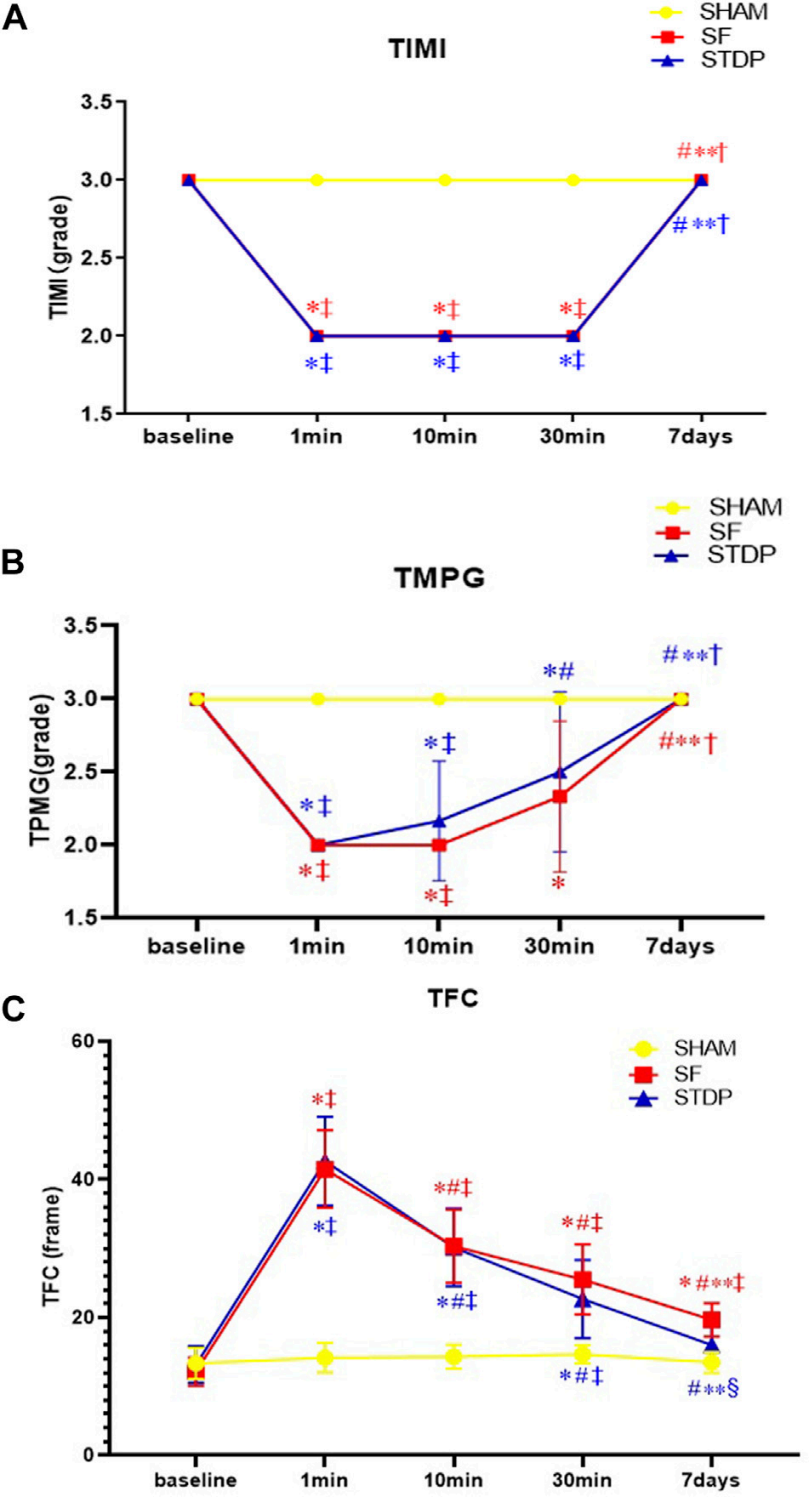
Line chart of LAD slow flow formation after coronary microembolism (Angiographic parameters at baseline, 1, 10, 30 min, and 7 days post LAD coronary microsphere injection). **(A–C)**: Line chart of angiographic parameters (TIMI, TMPG, and TFC) at baseline, 1, 10, 30 min, and 7 days post LAD coronary microsphere injection. Data are presented as the mean ± SD. *p* < 0.05 *vs. baseline, #vs. 1 min, **vs. 10 min, †vs. 30 min, ‡vs. Sham group, and §vs. SF group (*n* = 6 for each group).

The number of TFC frames formed at different time points is shown in Figure 3. In the SF group, the TFC frame was two times more than the SHAM group at the formation of slow blood flow. Coronary angiography was performed 7 days later; the TFC frame number did not fully recover to the baseline level. TMPG has been restored to level 3. There was no statistical difference in the recovery of coronary blood flow after 10 min of drug administration in the STDP group. But after 1 week, the TFC was significantly reduced in the STDP group compared with that in the SF group (Figure 3).

The videos about an image of LAD slow flow formation after coronary microembolism were also provided (submit multimedia files).

### Left Ventricular Angiography Examination

**FIGURE 4:**
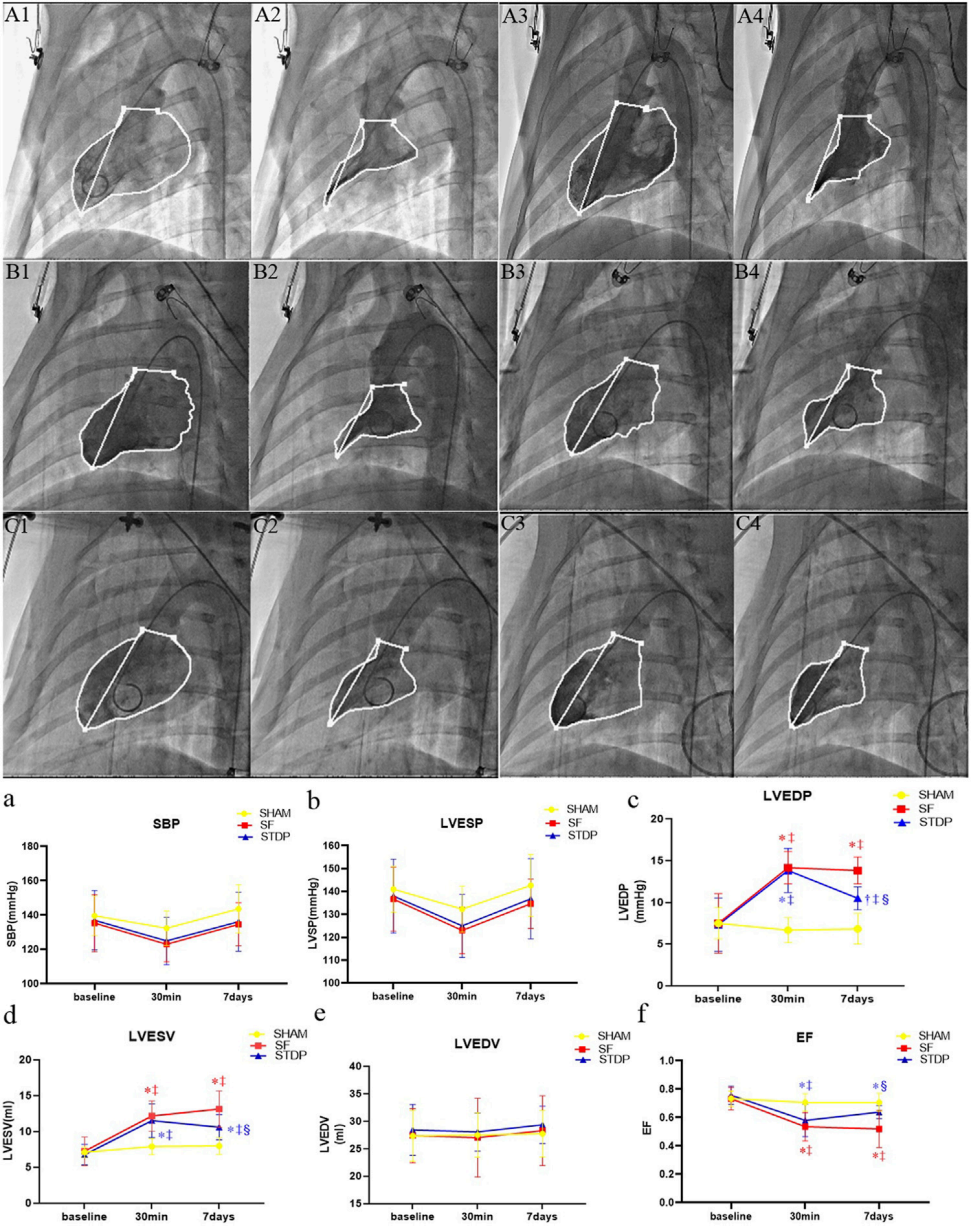
Hemodynamic parameters and cardiac function parameters at baseline, 30 min, and 7 days post LAD coronary microsphere injection. **(A–C)**: Left ventricular angiography baseline and 7 days after embolization in each group. **(A1,A2)**: Preoperative diastolic to systolic volume in the SHAM group. **(A3,A4)**: Postoperative 7 days diastolic to systolic volume in the SHAM group. **(B1,B2)**: Preoperative diastolic to systolic volume in the SF group. **(B3,B4)**: Postoperative 7 days diastolic to systolic volume in the SF group. **(C1,C2)**: Preoperative diastolic to systolic volume in the STDP group. **(C3,C4)**: Postoperative 7 days diastolic to systolic volume in the STDP group The activity of porcine myocardium decreased significantly after injection, and abnormal movement occurred during systole in the SF group 7 days after embolization. **(a–f)**: Line chart of hemodynamic parameters and cardiac function parameters at baseline, 30 min, and 7 days post LAD coronary microsphere injection. SBP, systolic pressure; LVESP, left ventricular end-systolic pressure; LVEDP, left ventricular end-dystolic pressure; LVESV, left ventricular end-systolic volume; LVEDV, left ventricular end-diastolic volume; LVEF, left ventricular ejection fraction. Data are presented as the mean ± SD. *p* < 0.05 *vs. baseline, †vs. 30 min, ‡vs. Sham group, and §vs. SF group (*n* = 6 for each group).

Left ventricular angiography measurement showed that the left ventricular EF decreased significantly at 30 min after microsphere injection, which was significantly higher at 7 days post embolization in the STDP group than that in the SF group (Figure 4f). Both left ventricular end-diastolic pressure and end-systolic volume were significantly lower, while EF was significantly higher in the STDP group than that in the SF group at 7 days post embolization (Figures 4c,d,f). SBP, left ventricle end-systolic pressure and end-diastolic volume had no significant change in the STDP group at 7 days post embolization (Figures 4a,b,e).

### Hematoxylin and Eosin Staining Results

**FIGURE 5:**
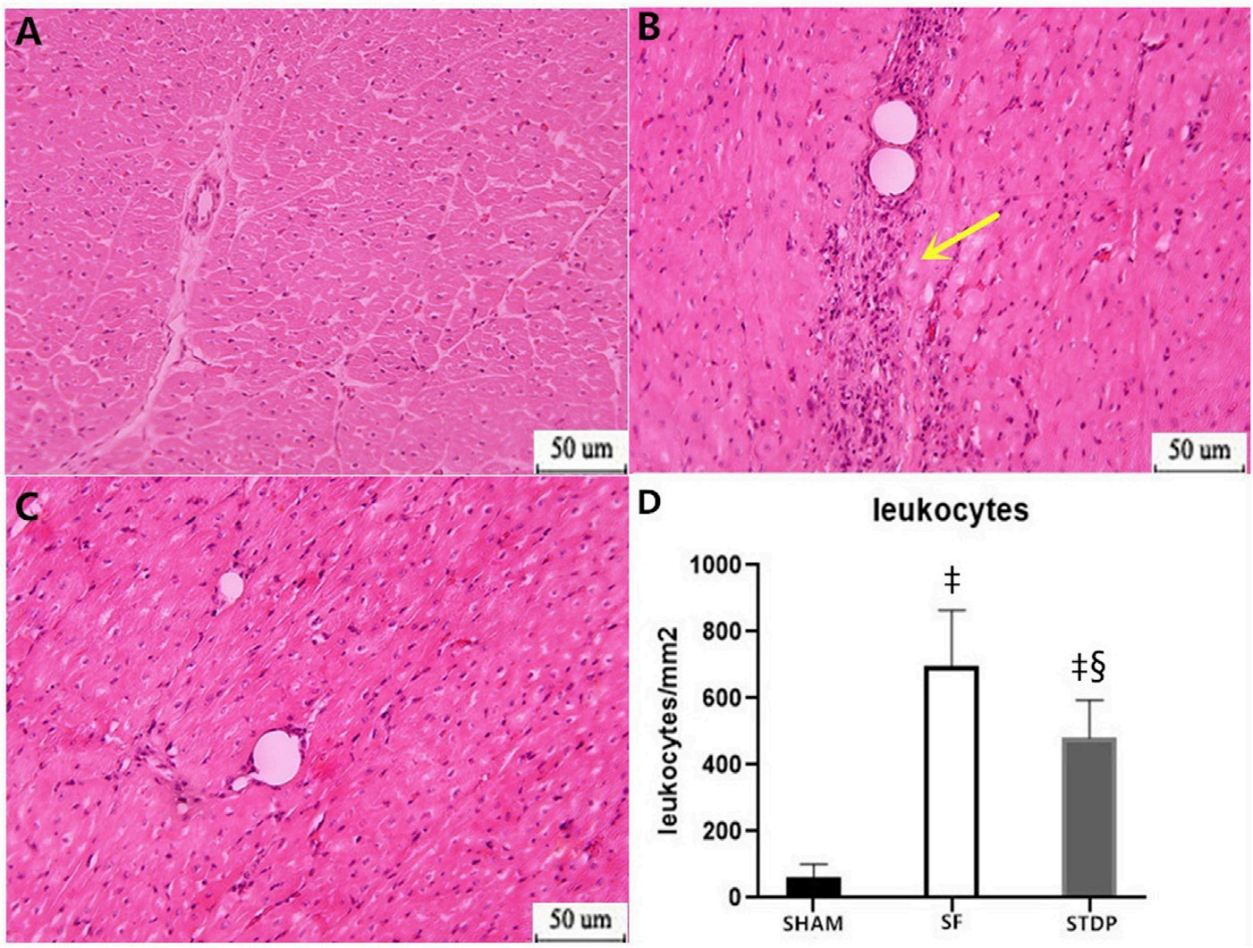
HE staining analysis of the myocardial specimen (myocardial infarction area) 7 days after operation. **(A)** SHAM group × 200: **(B)** SF group × 200; **(C)** STDP group × 200: **(D)** Quantified and showed results in the bar graph below the figure. In the SF group, inflammatory cell infiltration was obvious at the ischemia area. Inflammatory cell infiltration was also observed in the STDP group. Compared with the SF group, inflammatory cell infiltration decreased in the STDP group. Yellow arrows indicate a large infiltration of inflammatory cells. Data are presented as the mean ± SD. *p* < 0.05 ‡vs. Sham group, and §vs. SF group (*n* = 6 for each group).

Then, 7 days after microsphere injection, HE staining showed that leukocyte infiltration was significantly reduced in the STDP group than in the SF group (Figure 5).

### Immunohistochemical Staining Results

**FIGURE 6:**
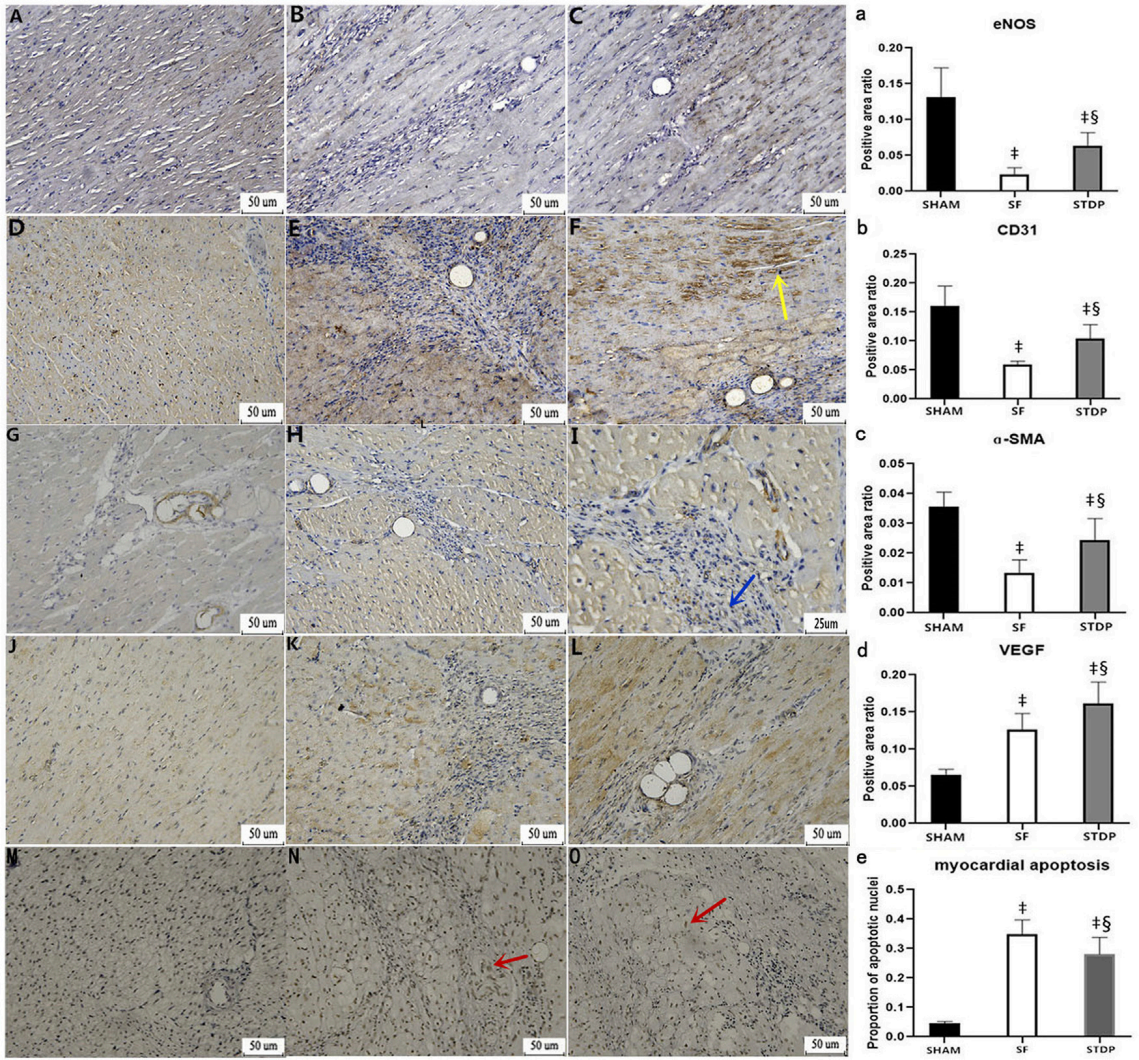
Immunohistochemical analysis of myocardial specimens. **(A–C)** eNOS expression: **(A)** SHAM group × 200; **(B)** SF group × 200; **(C)** STDP group × 200 **(D–F)** CD31 expression: **(D)** SHAM group × 200; **(E)** SF group × 200; **(F)** STDP group × 200 **(G–I)** ɑ-SMA expression: **(G)** SF group × 200; **(B)** STDF group × 200; **(C)** STDP group × 400 **(J–L)** VEGF expression: **(J)** SHAM group × 200; **(K)** SF group × 200; **(L)**: STDP group × 200 **(M–O)** Myocardial cell apoptosis staining: **(M)**: SHAM group × 200; **(N)**: SF group × 200; **(O)**: STDP group × 200. Yellow arrow: in the STDP group, the microvascular hyperplasia expressing CD31 was active in the ischemia area. Blue arrow: increased expression of ɑ-SMA at microarteries was observed in the ischemia area of the STDP group. Red arrows indicated the apoptotic nucleus. Brownish yellow granules appear in myocardial nuclei around the microembolization area (indicating the onset of myocardial cell apoptosis). **(a–e)**: quantified and showed results in the bar graph below the figure. Data are presented as the mean ± SD. *p* < 0.05 ‡vs. Sham group, and §vs. SF group (*n* = 6 for each group).

Heart samples were taken on the 7th day after the operation, and immunohistochemical staining was performed. Vascular smooth muscle cell motor protein-ɑ (ɑ-SMA): the vascular smooth muscle was stained brown and yellow by light microscopy and can be used to analyze the situation of new microvessels around the embolic area, CD31 staining reflects vascular endothelial density, and VEGF was an important factor modulating microvascular endothelial hyperplasia. eNOS (endothelium-induced NO synthase) activity reflects the function of microvascular endothelial relaxation. Figure 6 showed that the eNOS expression was weakened in the SF group and restored in the STDP group, especially in the area around blood vessels (Figures 6B–C,a). CD31 (Figures 6E–F,b) and ɑ-SMA (Figures 6G–I,c) changes were similar to those of eNOS. The expression of VEGF was increased at the embolic zone in the SF group, indicating compensated microvascular endothelial hyperplasia in this region post-embolism; this response was further enhanced in the STDP group (Figures 6K–L,d).

Heart samples were taken at 7th days after the operation, and myocardial cell apoptosis staining (EZClick™ TUNEL–in situ DNA Fragmentation/Apoptosis Assay Kit) was performed in the paraffin section. Under the light microscope, the number of brown-yellow particles and the apoptotic cardiomyocytes increased significantly in the SF group, which could be significantly reduced by STDP treatment (Figures 6N–O,e).

### Western Blot Results

**FIGURE 7:**
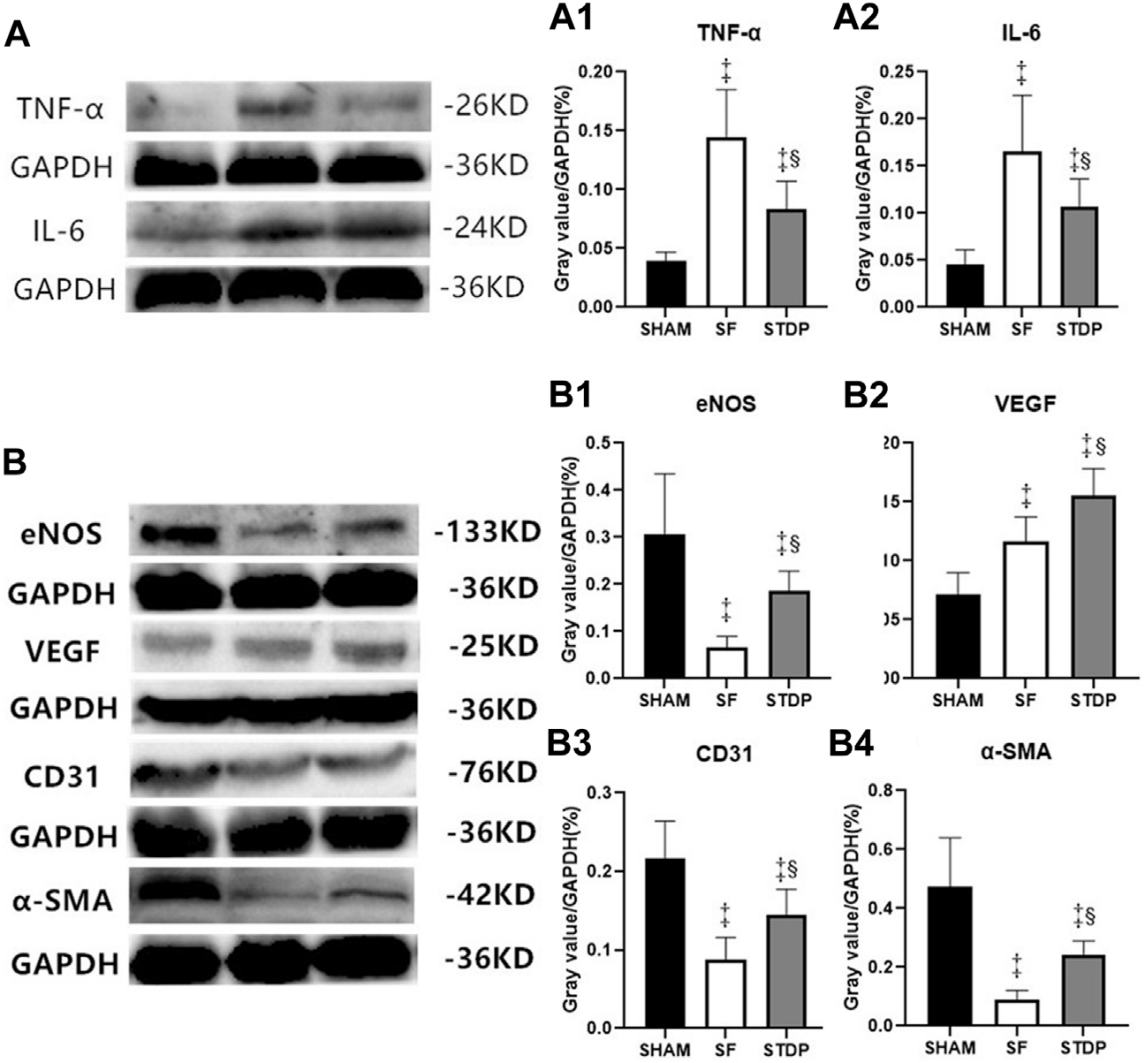
TNF-ɑ, IL-6, eNOS, VEGF, CD31, and ɑ-SMA in myocardial tissue of the ischemia area analyzed by Western blotting. **(A)** Expressions of TNF-ɑ and IL-6 7 days after operation **(A1)**: Ratio of TNF-ɑ/GAPDH in different groups. **(A2)**: Ratio of IL-6/GAPDH in different groups. **(B)** Expressions of eNOS, VEGF, CD31, and ɑ-SMA 7 days after operation. **(B1)**: Ratio of eNOS/GAPDH in different groups. **(B2)**: Ratio of VEGF/GAPDH in different groups. **(B3)**: Ratio of CD31/GAPDH in different groups. **(B4)**: Ratio of ɑ-SMA/GAPDH in different groups. Data are presented as the mean ± SD. *p* < 0.05 ‡vs. Sham group, and §vs. SF group (*n* = 6 for each group).

Western blotting analysis showed that the myocardial protein expression of inflammatory cytokines TNF-ɑ (Figures 7A,A1) and IL-6 (Figures 7A,A2) increased significantly in the SF group at 7th-day post-operation, which was significantly reduced in the STDP group. Western blotting analysis also showed that the myocardial protein expressions of eNOS, CD31, and ɑ-SMA in the embolic zone of the SF group were decreased, which was significantly upregulated in the STDP group (Figures 7B,B1–B4). Similar to immunohistochemical staining results, the myocardial protein VEGF expression was increased in the SF group and increased further in the STDP group (Figures 7B,B2).

## Discussion

After emergency PCI treatment, about 10%–30% of AMI patients still failed to achieve complete myocardial tissue perfusion because of slow flow or no flow in infarct-related vessels. Some patients still suffered from poor reperfusion at the myocardial level even after the TIMI III grade forward blood flow. Clinically, the short- and long-term prognosis after successful epicardial reperfusion therapy is poor, especially progressive deterioration of cardiac function or repeated ischemic cardiac events (Krug et al., 1966; Piana et al., 1994; Ito et al., 1996; Morishima et al., 2000; Iwakura et al., 2003). Its essence was myocardial microcirculation perfusion disorder. In the process of emergency PCI, both pieces of atheromatous plaque rupture and thrombosis particles fall off, or the atheromatous plaque of extrusion, patches of lipid debris falls off, matrix composition and endothelial cells, inflammatory cells, platelet and leukocyte adhesion to gather all, which can cause these ingredients to distal vascular and cause tiny blood vessels embolization: Coronary microembolism (CME). Current clinical intervention methods for CME: immediate application of sodium nitroprusside, thrombolysis, platelet GPIIb/IIIa receptor inhibitors, and other drugs, mechanical thrombolysis, and distal blood flow protection devices during coronary interventional therapy have not significantly improved the long-term prognosis of patients (Stone et al., 2005; Merla et al., 2007). Therefore, it is particularly important to screen out effective drugs for treating coronary slow blood flow. In this study, the intracoronary injection of the microsphere method was used to construct a miniature pig slow blood flow model to simulate the process of slow blood flow caused by the fragmentation of atherosclerotic plaque and the fall off of thrombus particles during PCI and to explore its pathophysiological mechanism and evaluate the effect of the Shexiang Tongxin dropping pill (STDP) on improving coronary slow blood flow, cardiac function, and possible mechanisms.

The major findings of the present experimental study are as follows: 1) STDP, a Chinese herbal drug with multi-component, multi-target, and multi-pathway pharmacological features, could improve TFC and cardiac function at 7 days post-operation in a swine model of SF. 2) Our results revealed that STDP could reduce myocardial infiltration of inflammatory cells and serum BNP and ET-1 levels while significantly increasing serum NO and NOS levels at 7 days post embolization. 3) Immunohistochemical staining and Western blot results also revealed reduced myocardial expressions of inflammatory cytokines (TNF-α, IL-6), while increased myocardial expressions of eNOS, VEGF, CD31, and α-SMA, TUNNEL results also demonstrated reduced myocardial apoptosis in the STDP group. Taken together, our results clearly demonstrated the results of STDP in this swine SF model and potential related mechanisms. To our best knowledge, this is the first report of STDP in the large animal SF model.

Our study showed that the improved TFC and cardiac function post-STDP might be linked with reduced inflammation responses and improved endothelial function and angiogenesis, as well as reduced myocardial apoptosis. STDP consisted of musk and toad crisp, Dan ginseng, artificial beef yellow, bear bile powder, and ice tablets; it is difficult to attribute the identical role of each component of the drug to observed net effects. As a whole, STDP promoted blood circulation, possibly by reducing the inflammatory responses and improving microcirculation *via* promoting angiogenesis and reducing endothelial dysfunction in this swine SF model. It is to note that future experimental studies are warranted to explore the causal relationship between the observed effects and various signaling pathways by observing the net effects of modulating related pathways suggested by this study, for example, pathways related to inflammation, endothelial function, and angiogenesis.

Preliminary small-scale clinical studies confirmed that STDP could improve angina pectoris and attenuate vascular endothelial dysfunction in patients with slow coronary flow (Wang et al., 2019; Lu et al., 2020; Tian et al., 2020); our data supplied experimental evidence and related mechanisms to explain the beneficial results on aforementioned clinical studies.

### Clinical Significance

This study highlights potential pharmacological mechanisms of STDP for improving coronary slow blood flow and cardiac function in this swine model of SF. Results from this study might contribute to the understanding of the pathophysiological mechanism of coronary microcirculation disorders and provide experimental evidence for the treatment of coronary slow flow with traditional Chinese medicine (TCM) such as STDP.

### Limitation

In this study, the coronary artery slow blood flow model was successfully prepared by intracoronary injection of microsphere suspension into LAD. However, the slow flow phenomenon caused by this method just simulated clinical myocardial infarction or percutaneous coronary intervention (percutaneous coronary intervention). The phenomenon of no re-flow/slow blood flow caused by plaque fragments and thrombus particles falling off to distal microvessels during PCI has a similar mechanism and was not entirely consistent with this animal model. Another study limitation is that we did not measure oxidative stress biomarkers in this study, which might be of importance in the interpretation of potential mechanisms for the observed beneficial effects of STDP in this model.

## Conclusion

In a word, the Shexiang Tongxin dropping pill, screened from the treasure house of Chinese medicine, serves as an effective drug for improving coronary microcirculation in the setting of SF. This study highlights potential pharmacological mechanisms of STDP for improving coronary slow blood flow and cardiac function in this swine model of SF; future studies are needed to validate the causal mechanism of STDP in the swine model of SF.

## Data Availability

The raw data supporting the conclusion of this article will be made available by the authors, without undue reservation.

## Supplementary Material

Supplementary Video A1 TFC and myocardial staining in the SHAM group before injection. TFC was normal (TIMI=3 and TMPG=3).

Supplementary Video A2 TFC and myocardial staining in the SHAM group 1 min after injection. TFC was normal (TIMI=3 and TMPG=3).

Supplementary Video A3 TFC and myocardial staining in the SHAM group 10 min after injection. TFC was normal (TIMI=3 and TMPG=3).

Supplementary Video A4 TFC and myocardial staining in the SHAM group 30 min after injection. TFC was normal (TIMI=3 and TMPG=3).

Supplementary Video A5 TFC and myocardial staining in the SHAM group 7 days after injection. TFC was normal (TIMI=3 and TMPG=3).

Supplementary Video B1 TFC and myocardial staining in the SF group before injection. TFC was normal (TIMI=3, TMPG=3).

Supplementary Video B2 TFC and myocardial staining in the SF group 1 min after injection (TIMI=2 and TMPG <3). TFC values were significantly increased compared with baseline (reached slow flow standard). The myocardial staining in the region dominated by the anterior descending branch was markedly weakened.

Supplementary Video B3 TFC and myocardial staining in the SF group 10 min after injection (TIMI=2 and TMPG<3).TFC in the SF group recovered significantly.

Supplementary Video B4 TFC and myocardial staining in the SF group 30 min after injection (TIMI=2 and TMPG<3).

Supplementary Video B5 TFC and myocardial staining in the SF group 7 days after injection (TIMI=3 and TMPG=3). TFC in the SF group further recovered 7 days after microsphere injection.

Supplementary Video C1 TFC and myocardial staining in the STDP group before injection. TFC was normal (TIMI=3 and TMPG=3).

Supplementary Video C2 TFC and myocardial staining in the STDP group 1 min after injection. (TIMI=2 and TMPG <3). TFC values were significantly increased compared with baseline (reach slow flow standard).

Supplementary Video C3 TFC and myocardial staining in the STDP group 10 min after administering a drug. (TIMI=2 and TMPG <3).Compared with the SF group, the TFC of the STDP group was improved after 10 min of administering drug, but there was no statistical difference.

Supplementary Video C4 TFC and myocardial staining in the STDP group 30 min after injection (TIMI=2 and TMPG<3).

Supplementary Video C5 TFC and myocardial staining in the STDP group 7 days after microsphere injection (TIMI=3 and TMPG =3). Compared with the SF group, the TFC of the STDP group was improved obviously on the 7^th^ day.

## Abbreviations

PCI, percutaneous coronary intervention; AMI, acute myocardial infarction; SHAM, sham-operated; SF, slow coronary flow; STDP, Shexiang Tongxin dropping pill; TCM, traditional Chinese medicine; TMPG, TIMI myocardial perfusion grade; TFC, TIMI frame count; CAG, coronary angiography; LAD, left anterior artery; LVSP, left ventricle systolic pressure; LVEDP, left ventricle end-diastolic pressure; LVESV, left ventricle end-systolic volume; LVEDV, left ventricle end-diastolic volume; LVEF, left ventricle ejection fraction; BNP, brain natriuretic peptide; TNI, troponin I; NO, nitric oxide; NOS, nitric oxide synthase; eNOS, endothelial nitric oxide synthase; VEGF, vascular endothelial growth factor; α-SMA, α-smooth muscle actin; TNF-α, tumor necrosis factor-alpha; IL-6, interleukin-6.
