# Supplementary material for: Efficacy of Shexiang Tongxin Dropping Pills in a Swine Model of Coronary Slow Flow
Source: Front Physiol. 2022 Jun 14;13:913399. doi: 10.3389/fphys.2022.913399 (PMC9239170; doi:10.3389/fphys.2022.913399)
Supplement: Supplementary file 3 [file Table1.DOCX]

**Supplementary Table 1**

**Table 1 TFC（frames） changes in pigs with experimental slow flow treated with various dosage of STDP in pilot study**

|  | SF | STDP 10mg/kg/d | STDP 20mg/kg/d | STDP 30mg/kg/d | STDP40mg/kg/d |
| --- | --- | --- | --- | --- | --- |
| baseline | 11 | 10 | 11 | 12 | 11 |
| 1 min | 42 | 46 | 44 | 45 | 43 |
| 10min | 35 | 34 | 32 | 32 | 31 |
| 30min | 30 | 28 | 28 | 27 | 28 |
| 7days | 22 | 20 | 19 | 16 | 16 |
