# Supplementary material for: Efficacy of Shexiang Tongxin Dropping Pills in a Swine Model of Coronary Slow Flow
Source: Front Physiol. 2022 Jun 14;13:913399. doi: 10.3389/fphys.2022.913399 (PMC9239170; doi:10.3389/fphys.2022.913399)
Supplement: Supplementary file 16 [file DataSheet1.docx]

**Appendix for supplementary ﬁle**

**Supplementary Video A**

A1-A5(SHAM group)

A1: TFC and myocardial staining in SHAM group before injection .TFC was normal (TIMI=3,TMPG=3).

A2:TFC and myocardial staining in SHAM group 1min after injection.TFC was normal (TIMI=3,TMPG=3).

A3:TFC and myocardial staining in SHAM group 10min after injection.TFC was normal (TIMI=3,TMPG=3).

A4:TFC and myocardial staining in SHAM group 30min after injection.TFC was normal (TIMI=3,TMPG=3).

A5:TFC and myocardial staining in SHAM group 7days after injection.TFC was normal (TIMI=3,TMPG=3).

**Supplementary Video B**

B1-B5(SF group)

B1: TFC and myocardial staining in SF group before injection .TFC was normal (TIMI=3,TMPG=3).

B2: TFC and myocardial staining in SF group 1min after injection(TIMI=2,TMPG<3).

TFC values were significantly increased compared with baseline（reach slow flow standard）, The myocardial staining in the region dominated by the anterior descending branch was markedly weakened.

B3: TFC and myocardial staining in SF group 10 min after injection(TIMI=2,TMPG<3).

TFC in SF group recovered significantly .

B4: TFC and myocardial staining in SF group 30 min after injection(TIMI=2,TMPG<3).

B5: TFC and myocardial staining in SF group 7days after injection(TIMI=3,TMPG=3).

TFC in SF group further recovered 7days after microsphere injection.

**Supplementary Video C**

C1-C5（STDP group）

C1: TFC and myocardial staining in STDP group before injection .TFC was normal (TIMI=3,TMPG=3).

C2: TFC and myocardial staining in STDP group 1min after injection.(TIMI=2, TMPG <3).

TFC values were significantly increased compared with baseline（reach slow flow standard).

C3: TFC and myocardial staining in STDP group 10 min after administering drug.(TIMI=2, TMPG <3).

Compared with the SF group,The TFC of STDP group was improved after 10 min of administering drug, but was no statistical difference.

C4:TFC and myocardial staining in STDP group 30 min after injection(TIMI=2,TMPG<3).

C5: TFC and myocardial staining in STDP group 7days after microsphere injection(TIMI=3, TMPG =3)

Compared with the SF group, the TFC of STDP group was improved obviously in 7 days.
